# Value of threshold growth for the diagnosis of hepatocellular carcinoma using LI-RADS

**DOI:** 10.1186/s40644-025-00902-z

**Published:** 2025-07-01

**Authors:** Jae Seok Bae, Jeong Min Lee, Jeong Hee Yoon, Jae Hyun Kim, Sun Kyung Jeon, Jeongin Yoo

**Affiliations:** 1 Department of Radiology, Seoul National University Hospital, 101 Daehak-ro, Jongno-gu, Seoul, 03080 Republic of Korea; 2 Department of Radiology, Seoul National University College of Medicine, 103 Daehak-ro, Jongno-gu, Seoul, 03080 Republic of Korea; 3 Institute of Radiation Medicine, Seoul National University Medical Research Center, 103 Daehak-ro, Jongno-gu, Seoul, 03080 Republic of Korea

**Keywords:** Carcinoma, hepatocellular, Diagnostic imaging, Liver imaging reporting and data system, Liver transplantation, Threshold growth

## Abstract

**Background:**

The utility of threshold growth (TG) in hepatocellular carcinoma (HCC) imaging remains contentious across major guidelines. This study aimed to investigate the diagnostic implications of TG in HCC diagnosis using the criteria set by the Liver Imaging Reporting and Data System (LI-RADS).

**Methods:**

In this single-center retrospective study, three radiologists independently evaluated pre-transplantation hepatobiliary agent-enhanced MR images and prior CT/MR images using LI-RADS v2018 in consecutive patients who underwent liver transplantation between January 2010 and November 2022. TG was defined as a ≥ 50% size increase in ≤ 6 months. Explanted livers served as reference standards. Frequencies of TG between HCCs and non-HCCs were compared using Fisher’s exact test, and interobserver agreement was assessed using Fleiss κ statistics. The diagnostic performance of LI-RADS category 5 in the diagnosis of HCC was assessed with and without considering TG as a major feature. McNemar tests were used to compare results.

**Results:**

The cohort included 158 patients (mean age, 59.1 ± 7.5 years; 130 males) with 280 observations (207 HCCs, 5 non-HCC malignancies, and 68 benign lesions). TG was identified in 44 (15.7%) observations. Interobserver agreement on TG was moderate (κ = 0.280). Incorporating TG as a major feature significantly enhanced the sensitivity of LI-RADS category 5 in diagnosing HCC (33.8% vs. 40.6%, *p* < 0.001) without compromising specificity (100.0% vs. 94.5%, *p* = 0.125).

**Conclusions:**

Incorporating TG as a major criterion in LI-RADS category 5 enhanced the diagnostic sensitivity for HCC in liver transplant candidates with minimal impact on specificity. However, TG demonstrated a variable interobserver agreement.

**Trial registration:**

Not applicable.

**Supplementary Information:**

The online version contains supplementary material available at 10.1186/s40644-025-00902-z.

## Background

Threshold growth (TG)—a major feature of the Liver Imaging Reporting and Data System (LI-RADS)—is defined as size increase by ≥ 50% in ≤ 6 months [1]. In theory, however, TG itself is not specific for hepatocellular carcinoma (HCC); it can be observed in non-HCC malignancies or even benign lesions because cells other than hepatocytes can also proliferate. Indeed, TG has been reported in non-HCC malignancies and benign lesions [2]. Considering this limitation, TG is only an ancillary feature in the Korean Liver Cancer Association-National Cancer Center (KLCA-NCC) guideline and is not recognized in the European Association for the Study of the Liver (EASL) guideline [3, 4]. A recent individual patient data meta-analysis study reported that among the LI-RADS major imaging features, including non-rim arterial phase hyperenhancement (APHE), non-peripheral washout, and enhancing capsule, only TG was not associated with HCC [5]. This finding underscores the need to reassess the incorporation of TG as a major feature in LI-RADS, particularly because it aligns with the Organ Procurement and Transplantation Network diagnostic criteria for liver transplantation (LT) and reflects broader malignancy characteristics rather than being specific to HCC. Consequently, the literature presents conflicting findings on the diagnostic value of TG for HCC in a mixed surgical population comprising both hepatic resection and LT candidates [2, 6–9]. However, the diagnostic role of TG should be evaluated by focusing on the LT candidates in whom LI-RADS has been intended to be most clinically relevant [1]. Therefore, this study aimed to evaluate the diagnostic utility of TG in identifying HCC among LT candidates, employing hepatobiliary agent-enhanced MRI (HBA-MRI) scanning according to the LI-RADS criteria.

## Methods

This retrospective study was approved by the institutional review board of our hospital (IRB No. H-2403-048-1519), and the requirement for informed consent was waived.

### Patients

**Fig. 1:**
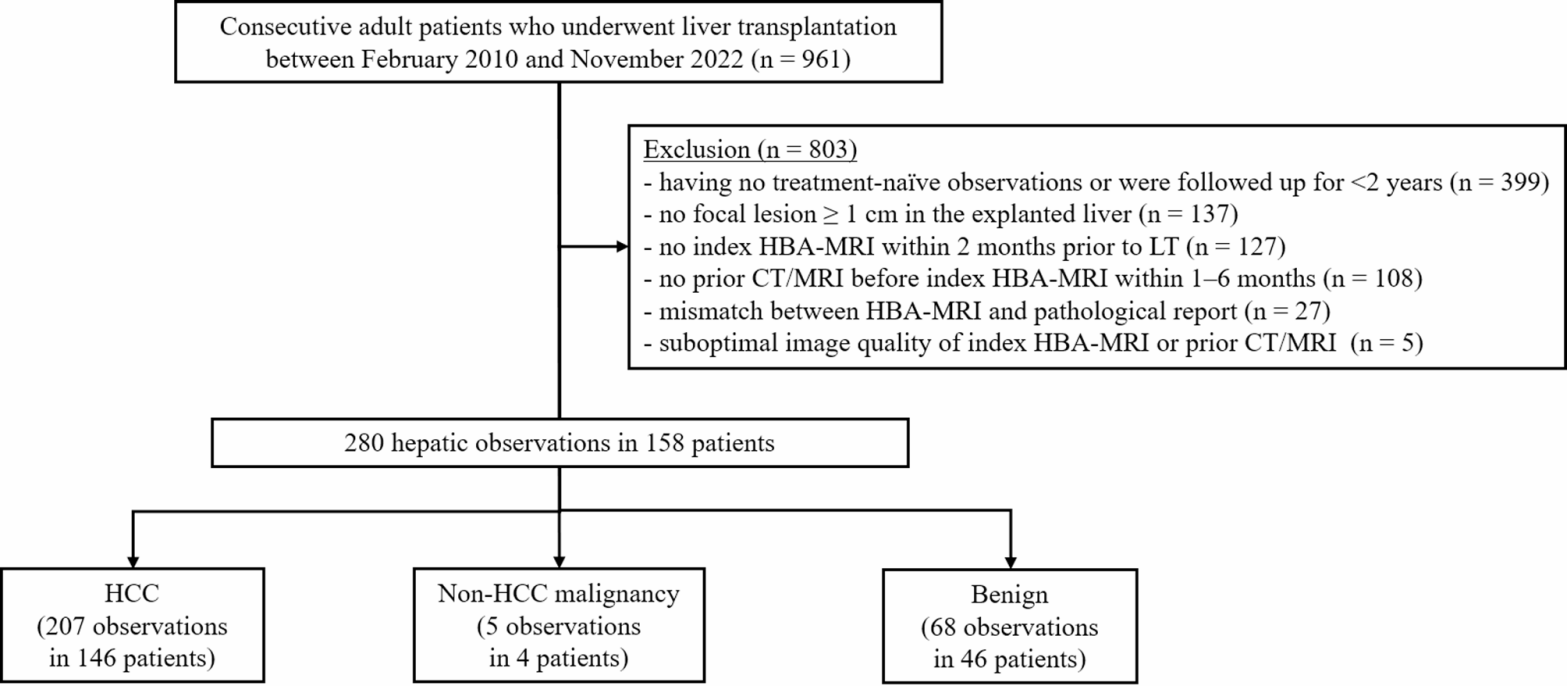
Study flow diagram

From a referral hospital, we retrospectively identified consecutive adult patients who underwent LT due to HCC between February 2010 and November 2022. A study coordinator (board-certified radiologist with 11 years of experience in abdominal imaging) reviewed electronic medical records to find eligible patients by applying the following exclusion criteria: (a) having no treatment-naïve observations or were followed up for < 2 years (see *Reference standards*), (b) no focal lesion ≥ 1.0 cm in the explanted liver, (c) no index HBA-MRI scan within 2 months prior to LT, (d) no prior CT/MRI scan before the index HBA-MRI within 1–6 months, (e) mismatch between HBA-MRI results and pathological report (e.g. a 3 cm-sized HCC in liver segment 8 on HBA-MRI but in liver segment 6 on the pathological report), and (f) suboptimal image quality of index HBA-MRI or prior CT/MRI examination including failure to meet the LI-RADS requirements (Fig. 1). Patients who had undergone prior CT before HBA-MRI scans within 1 month were excluded because a significant number of them had undergone HBA-MRI for additional characterization subsequent to prior CT, and a 1-month interval may not be sufficient for assessing TG [6, 8]. All included patients were in the LI-RADS target population (i.e., any cause of cirrhosis or chronic hepatitis B) [1]. Among the final 158 patients, 80 had been included in previous studies [10, 11]. However, previous studies assessed the diagnostic performance of the LR-TIV category and LR-TR algorithm, respectively, whereas our current study aimed to specifically assess the diagnostic role of TG in the diagnosis of HCC using HBA-MRI in LT candidates.

### HBA-MRI examination

All HBA-MRI examinations and previous imaging examinations evaluated in our study met the standards of LI-RADS version 2018. Imaging protocols and scan parameters of HBA-MRI are described in Additional file 1 (Supplementary methods and Table S1).

### Image analysis

Before image review, the study coordinator reviewed the index HBA-MR images to identify treatment-naïve observations. The identified observations on HBA-MRI were then annotated for independent review by three board-certified radiologists (J.H.K., S.K.J., and J.Y. with 11, 10, and 10 years of experience in abdominal imaging, respectively). The reviewers were informed that the patients had undergone LT but were blinded to the pathological information. The reviewers assessed the observations according to LI-RADS v2018 and assigned LI-RADS categories. In addition, the reviewers were provided with prior CT or MR images to measure the size of the observations to assess growth. Observation growth was evaluated by measuring and contrasting the maximum diameter of the observation on the index HBA-MRI with prior CT or MR images. The reviewers measured the observation size during the phase exhibiting the clearest margins, such as hepatobiliary phase (HBP) or transitional phase (TP) on MRI and portal venous phase (PVP) or delayed phase (DP) on CT. Sizes were compared during the same phase and on the same plane between the index HBA-MRI and prior CT or MR images. Lesion growth was categorized using LI-RADS v2018 criteria, distinguishing between TG and subthreshold growth (new lesion of any size or < 50% diameter increase in ≤ 6 months).

### Reference standards

For pathological assessment, we referred to the routine pathological reports of the liver explants made by hepatobiliary-specialized pathologists. When suspicious lesions were identified on gross examination, microscopic evaluation using hematoxylin and eosin-stained slides were performed. In addition to the pathological diagnosis of the tumor, its size and location were retrieved.

Among the 280 observations, 236 were confirmed by pathology: 207 HCCs, four cHCC-CCAs (combined hepatocellular carcinoma-cholangiocarcinomas), one iCCA (intrahepatic cholangiocarcinoma), 14 dysplastic nodules, and 10 focal nodular hyperplasia-like nodules. The remaining 44 lesions were noninvasively diagnosed using common imaging characteristics and regression or stability over a period of ≥ 2 years: 43 regenerative or dysplastic nodules and one hemangioma.

### Statistical analysis

Continuous variables are shown as mean ± standard deviation or median values (ranges), and categorical variables are shown as numbers (percentages), as appropriate. The independent t-test or Mann–Whitney U test was used to compare continuous variables, and the chi-square test or Fisher exact test was used to compare categorical variables. The sensitivity, specificity, positive predictive value, and negative predictive value of LR-5 category for diagnosing HCC with or without consideration of TG as the major imaging feature were calculated. Interobserver agreement for the percentages of interval growth was assessed using intraclass correlation coefficient. Interobserver agreement for the imaging features including TG and LI-RADS category were assessed using Fleiss κ statistics. The following convention was used to interpret κ values: <0.20, poor; 0.21–0.40, fair; 0.41–0.60, moderate; 0.61–0.80, substantial; and 0.81–1.00, nearly perfect [12]. Statistical analyses were performed using commercially available software (MedCalc, version 19.0.7, MedCalc Software; IBM released 2020; SPSS Statistics for Windows, Version 27.0, IBM, NY, USA). A two-sided *p* < 0.05 was considered to indicate statistical significance.

## Results

### Patients

A total of 158 patients (age, 59.1 ± 7.5 years; 130 men) with 280 observations including 207 HCCs were included (Table 1). The most common etiology for chronic liver disease was hepatitis B viral infection (72.8% [115/158]), followed by hepatitis C viral infection (10.8% [17/158]). Most patients had cirrhosis on pathology (84.8% [134/158]). Among the 158 patients, 85 patients had a single observation, and 73 patients had multiple observations. The median tumor size was 1.5 cm for HCC, 3.0 cm for non-HCC malignancies, and 1.5 cm for benign lesions.

**Table 1:** Patient and observation characteristics

Characteristics	Value
Age, years	59 (54–64)
Sex (male: female)	130: 28
Cause of underlying liver disease, n (%)	
Hepatitis B virus	115 (72.8)
Hepatitis C virus	17 (10.8)
Alcohol	12 (7.6)
Idiopathic	8 (5.1)
Others	6 (3.8)
Cirrhosis on pathology, n (%)	134 (84.8)
Child-Pugh class, n (%)	
A	66 (41.8)
B	75 (47.5)
C	17 (10.8)
Laboratory findings	
Albumin (g/dl)	3.3 (2.9–3.7)
Total bilirubin (g/dl)	2.0 (1.1–3.4)
PT-INR	1.4 (1.2–1.7)
AFP (ng/ml)	8.4 (3.7–33.8)
PIVKA-II (AU/ml)	28 (19–86)
Number of observations per patient, n (%)	
1	85 (53.8)
2	43 (27.2)
3	23 (14.6)
4	5 (3.2)
5	2 (1.3)
Lesion size (cm)	
HCC	1.5 (1.2–2.2)
Non-HCC malignancy	3 (2.2–5.1)
Benign	1.5 (1.1–1.9)
Pathologic diagnosis, n (%)	
HCC	207 (73.9)
Non-HCC malignancy	5 (1.8)
Benign	68 (24.3)

Unless otherwise indicated, continuous variables are reported as median (interquartile range) and categorical variables as number (%)

AFP, alpha-fetoprotein; HCC, hepatocellular carcinoma; IQR, interquartile range; PIVKA-II, protein induced by vitamin K absence or antagonist II; PT-INR, prothrombin time–international normalized ratio

### Interval change in the size of hepatic observations

Among the 280 observations, 219 (78.2%) observations showed interval growth (TG and subthreshold growth) and 61 (21.8%) showed no interval growth. TG was noted in 44 (15.7%) of the total 280 observations: 3 (8.3%) of 36 observations at 1–2 months, 9 (15.0%) of 60 at 2–3 months, 11 (16.9%) of 65 at 3–4 months, 10 (18.5%) of 54 at 4–5 months, and 11 (16.9%) of 65 at 5–6 months.

The size of the hepatic observations was measured on prior CT (83.6%) and MRI (16.4%) scans. The frequency of TG was not significantly different between the prior imaging modalities (17.1% vs. 8.7%, *p* = 0.187). Of the 234 observations evaluated using prior CT scans, the size was measured on DP for 174 observations (74.4%) and PVP for 33 observations (14.1%). The remaining 27 observations were not visible on prior CT scans. Of the 46 observations evaluated using prior MRI, the size was measured on HBP for 44 observations (95.7%) and PVP for two (4.4%) observations. There was no significant difference in the frequency of TG according to the phase used in the prior CT/MRI scans (all *p* ≥ 0.339).

TG was observed in 37 of the 207 (17.9%) HCCs, one of the five (20.0%) non-HCC malignancies, and six of the 68 (8.8%) benign conditions (Table 2). There was no significant difference in the frequency of TG among HCCs, non-HCC malignancies, and benign lesions (*p* = 0.198).

**Table 2:** Interval growth rate according to pathological diagnosis

	HCC (*n* = 207)	Non-HCC malignancy (*n* = 5)	Benign (*n* = 68)
No growth	45 (21.7%)	1 (20.0%)	15 (22.1%)
Subthreshold growth			
Growth < 50%	112 (54.1%)	3 (60.0%)	33 (48.5%)
New lesion	13 (6.3%)		14 (20.6%)
Threshold growth	37 (17.9%)	1 (20.0%)	6 (8.8%)

Unless otherwise indicated, continuous variables are reported as median (interquartile range) and categorical variables as number (%).

HCC, hepatocellular carcinoma.

Interobserver agreement for the percentage of growth was moderate (intraclass correlation coefficient, 0.469; 95% CI, 0.387–0.548). Interobserver agreement for TG was fair (κ = 0.266; 95% CI, 0.198–0.333), whereas other major features including non-peripheral APHE, non-peripheral washout, and enhancing capsule showed κ values of 0.738 (95% CI, 0.671–0.806), 0.475 (95% CI, 0.408–0.543), and 0.353 (95% CI, 0.286–0.421), respectively.

The distributions of interval growth rates according to LI-RADS categories are presented in Additional file 1 (Table S2). TG was observed in 5.6% (1/18), 3.3% (5/152), 36.4% (32/88), and 31.6% (6/19) of the LR-3, LR-4, LR-5, and LR-M lesions, respectively.

### Effect of TG on the diagnostic performance of LI-RADS

**Fig. 2:**
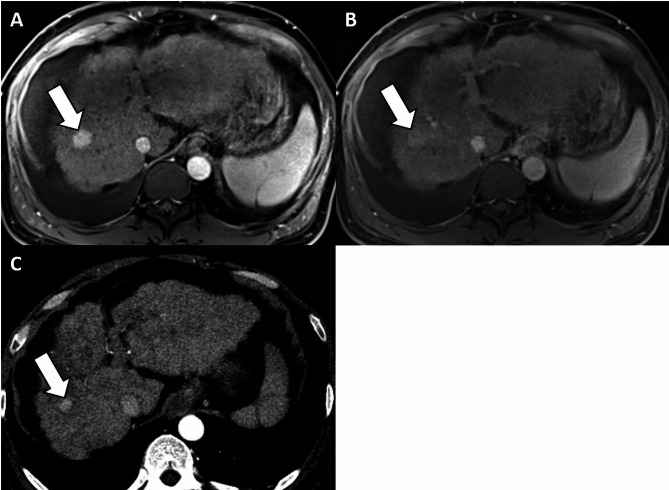
Images of typical threshold growth in a 47-year-old man with hepatocellular carcinoma. **(a)** Arterial phase hepatobiliary contrast agent-enhanced axial MRI scan shows an 18 mm observation with non-rim hyperenhancement in segment 8 of the liver (arrow). **(b)** Portal venous phase axial MR image shows no washout or enhancing capsule (arrow). Accordingly, this observation was categorized as LR-4. However, the same observation measured 11 mm on an arterial phase axial CT image obtained 4 months before **(c)**, corresponding to threshold growth. Considering the presence of additional major features of threshold growth, the observation was recategorized as LR-5. Hepatocellular carcinoma was confirmed in the explanted liver. CT, computed tomography; MRI, magnetic resource imaging

**Fig. 3:**
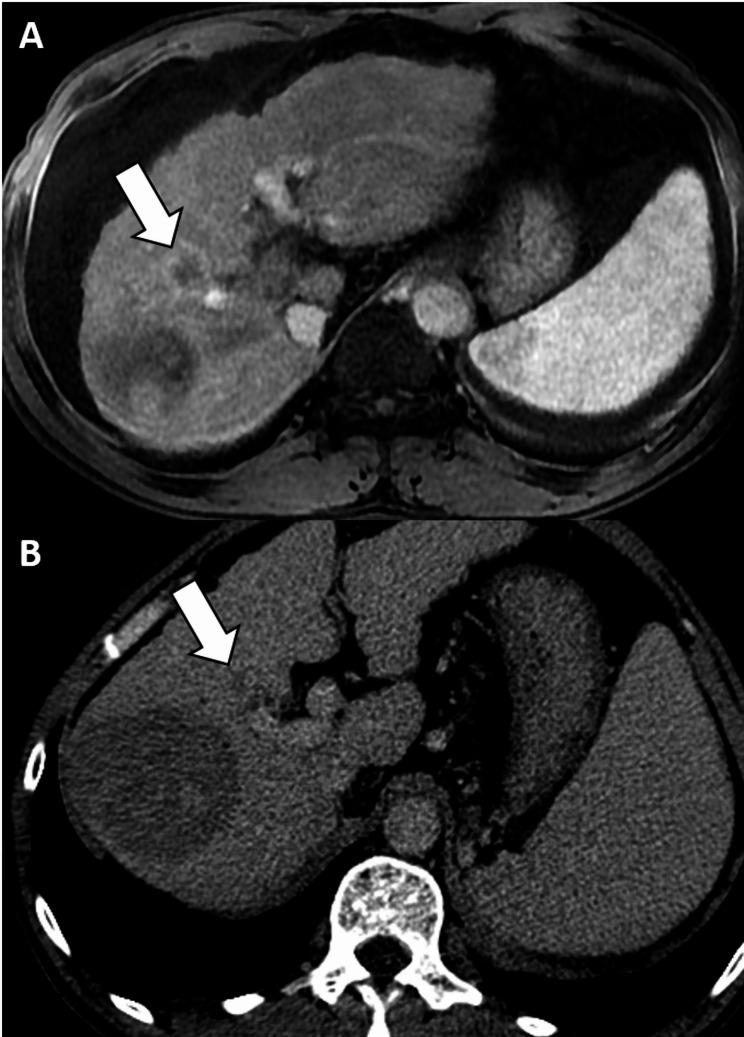
Nonspecific nature of threshold growth. **(a)** Arterial phase hepatobiliary contrast agent-enhanced axial MRI scan shows a 23 mm observation with rim hyperenhancement in segment 8 of the liver (arrow), which corresponds to LR-M. The same observation measured 15 mm on a delayed phase axial CT image obtained 5 months before **(b)**, corresponding to threshold growth. There was no modification of LI-RADS category because the observation was initially categorized as LR-M. After liver explantation, cholangiocarcinoma was confirmed. Furthermore, there was a larger ablation zone at the posterolateral aspect of the observation on CT and MR images. CT, computed tomography; LI-RADS, Liver Imaging Reporting and Data System; MRI, magnetic resource imaging

Prior to the inclusion of TG as a major feature, the 37 lesions in the HCC group were categorized as LR-3 (*n* = 2), LR-4 (*n* = 17), LR-5 (*n* = 14), and LR-M (*n* = 4). When TG was included, two LR-3 and 12 LR-4 lesions were categorized as LR-5 (Fig. 2). The one non-HCC malignancy was LR-M and the inclusion of TG as a major feature did not alter its LI-RADS category (Fig. 3). The six benign conditions that showed TG were categorized as LR-3 (*n* = 2), LR-4 (*n* = 3), and LR-M (*n* = 1). When TG was included, one of the LR-3 lesions (focal nodular hyperplasia-like nodule) and three LR-4 lesions (two dysplastic nodules and a focal nodular hyperplasia-like nodule) were categorized as LR-5, resulting in four false-positive results.

When TG was not used as a major feature, the sensitivity and specificity of LR-5 were 33.8% and 100.0%, respectively (Table 3). When TG was used as a major feature, the sensitivity of LR-5 significantly increased (33.8% vs. 40.6%, *p* < 0.001), whereas specificity was similar (100.0% vs. 94.5%, *p* = 0.125). However, for LR-4 and LR-5 combined, the inclusion of TG as a major feature significantly increased sensitivity (83.6% vs. 90.3%, *p* < 0.001) and decreased specificity (46.6% vs. 27.4%, *p* < 0.001) (Table 3).

**Table 3:** Diagnostic performance of LI-RADS for the diagnosis of HCC with or without TG

	Sensitivity (95% CI)	Difference	*P* value	Specificity (95% CI)	Difference	*P* value
LR-5						
without TG	33.8 (27.4, 40.7)	6.8 (3.3, 10.2)	< 0.001	100.0 (95.1, 100.0)	-5.5 (-10.7, -0.3)	0.125
with TG	40.6 (33.8, 47.6)			94.5 (86.6, 98.5)		
LR-4 and LR-5						
without TG	83.6 (77.8, 88.3)	6.8 (3.3, 10.2)	< 0.001	46.6 (34.8, 58.6)	-19.2 (-28.2, 10.2)	< 0.001
with TG	90.3 (85.5, 94.0)			27.4 (17.6, 39.1)		

Data are reported as percentages (95% CI).

CI, confidence interval; HCC, hepatocellular carcinoma; LI-RADS, Liver Imaging Reporting and Data System; LR-4, LI-RADS category 4; LR-5, LI-RADS category 5; TG, threshold growth.

In the subgroup analysis, the sensitivity and specificity of LI-RADS LR-5 with TG were 36.5% and 92.7% for observations ≤ 2.0 cm, respectively, and 54.2% and 100.0% for observations > 2.0 cm, respectively (Additional file 1, Table S3). The use of TG as a major feature significantly increased the sensitivity of LR-5 for observations ≤ 2.0 cm (28.3% vs. 36.5%, *p* < 0.001) without significant loss of specificity (100.0% vs. 92.7%, *p* = 0.125).

## Discussion

Although TG is a major feature of LI-RADS, it is not regarded as such by other major guidelines from KLCA-NCC or EASL, as interval growth is not specific to HCC and can be observed in non-HCC malignancies or even benign entities [2–4]. Consequently, the value of TG for diagnosing HCC has been controversial in the literature [2, 6–9]. To address this, we evaluated the diagnostic utility of TG in identifying HCC among 158 LT candidates using HBA-MRI. We found that incorporating TG as a major feature significantly increased the sensitivity of LR-5 while maintaining its specificity, consistent with the findings of a recent study by Choi et al. [6]. Although Park et al. previously reported that TG is not a significant diagnostic indicator of HCC, their study population primarily consisted of surgical resection patients rather than LT candidates, with only 3.1% of patients undergoing LT [8]. In contrast, our study population consisted of LT candidates, who have a higher pretest probability of HCC. Diagnostic criteria aimed at increasing sensitivity tend to perform well in this population, despite the risk of reducing specificity [13]. This difference in study populations may explain the discrepancy. We believe that the value of LI-RADS imaging features should be evaluated in LT candidates, for whom LI-RADS was intended.

Notably, our results differ from the findings of a recent meta-analysis, which found no significant association between TG and HCC [5]. However, methodological differences may explain this discrepancy. Specifically, four of the five included studies primarily utilized composite reference standards combining pathology and imaging, with liver explantation performed in only 12.5–48.9% of lesions and a significantly lower HBV prevalence (3.9–21.9%) compared to our study (72.8%) [14–17]. In contrast, our study exclusively employed explanted livers as the solid pathological reference standard for all lesions, providing robust diagnostic verification. Additionally, the study by Seo et al., although exclusively conducted on LT candidates and with a higher HBV prevalence (53.7%), utilized CT imaging and applied LI-RADS v2014 or OPTN/UNOS definitions, differing markedly from our use of LI-RADS v2018 with gadoxetic acid-enhanced MRI [18]. These differences in study design, imaging modalities, patient populations, and diagnostic criteria highlight the necessity for cautious interpretation when comparing studies, underscoring the distinct strength of our study’s homogeneous reference standard.

Incorporation of TG as a major feature aided the diagnosis of 14 additional HCCs (12 LR-4 and two LR-3 observations) while leading to the misclassification of four benign lesions (three LR-4 and one LR-3 observations) as HCCs. LR-4 observations sized 1.0–1.9 cm with non-rim APHE and an enhancing capsule, or sized ≥ 2.0 cm with non-rim APHE but not an enhancing capsule or non-peripheral washout, can be upgraded to LR-5 by including TG [1]. In our study, 80% of LR-4 observations with corresponding imaging features were HCC, significantly contributing to the increased sensitivity. For LR-3 observations to be upgraded to LR-5 by including TG, the observations sized 1.0–1.9 cm should have non-rim APHE but not an enhancing capsule or non-peripheral washout [1]. In our study, three LR-3 observations demonstrated these imaging features, and 2/3 of them were HCC. However, focal nodular hyperplasia-like nodules or nodular arterioportal shunts showing TG could be misclassified as LR-5 instead of LR-3. Therefore, upgrading LR-3 observations to LR-5 by including TG should be done cautiously, given the significant clinical implications between these categories.

Regarding tumor size, TG had a different impact on smaller (≤ 2.0 cm) vs. larger (> 2.0 cm) observations: the sensitivity of the LR-5 category for diagnosing HCC significantly increased in smaller observations but not in larger ones. This result aligns with findings by Choi et al., although their study used a size criterion of 3.0 cm [6]. This discrepancy in the diagnostic impact of TG based on a tumor size of 2.0 cm can be explained by the LI-RADS CT/MRI diagnostic algorithm. Observations < 2.0 cm can be upgraded to LR-5 by including TG if they have non-rim APHE but not an enhancing capsule or non-peripheral washout (LR-3) or non-rim APHE and an enhancing capsule (LR-4). In contrast, observations ≥ 2.0 cm should have non-rim APHE but not an enhancing capsule or non-peripheral washout (LR-4) to be upgraded to LR-5 by including TG. Smaller LR-4 observations show the additional major feature of an enhancing capsule compared to larger LR-4 observations, suggesting they are more likely to be HCCs. Thus, including TG is helpful for diagnosing HCC during surveillance of patients on an LT waiting list.

Given the nonspecific nature of TG for HCC, there is a risk of losing specificity with its inclusion as a major feature, despite the significant increase in sensitivity for diagnosing HCC. For instance, any LR-4 observation ≥ 1.0 cm with non-rim APHE but not an enhancing capsule or non-peripheral washout can be upgraded to LR-5 with the inclusion of TG. This could result in focal nodular hyperplasia-like nodules being categorized as LR-5, thereby decreasing specificity. The risk of losing specificity becomes even greater when combining LR-4 and LR-5, as demonstrated in our study. LR-3 observations that can be upgraded to LR-4 with the inclusion of TG include those < 1.0 cm with non-rim APHE only but not an enhancing capsule or non-peripheral washout, < 2.0 cm with an enhancing capsule or non-peripheral washout but not non-rim APHE, and ≥ 2.0 cm without an enhancing capsule, non-peripheral washout, and non-rim APHE. These observations may be focal nodular hyperplasia-like nodules or cirrhosis-associated nodules. Given the potential for over-diagnosis, careful consideration is essential when upgrading the classification of LR-4 or LR-3 lesions to avoid unnecessary treatments or follow-up procedures. Considering this, prudent deliberation is necessary for future revisions of the KLCA-NCC guidelines, which currently include interval growth as an ancillary feature.

This study has some limitations. First, this is a single-institutional retrospective study and is, therefore, subject to selection bias. Second, there was a small number of patients with non-HCC malignancies (*n* = 5) and benign lesions (*n* = 68), limiting the evaluation of specificity. The small number of observations demonstrated TG (*n* = 44) is another limitation. Third, focusing on LT candidates with hepatitis B viral infection may limit the extrapolation of our findings to the broader population at risk for HCC (i.e., all patients with chronic hepatitis B or liver cirrhosis of any cause). Additionally, LT candidates typically exhibit distinct clinical profiles and disease progression which may not reflect the broader patient population, potentially limiting the generalizability of our results.

## Conclusions

This study underscores the importance of TG in improving the diagnostic sensitivity of LI-RADS for HCC in LT candidates, although its practical use is limited by a slight yet clinically meaningful decrease in specificity and modest interobserver agreement. Further validation in broader populations and improvement in interobserver consistency are warranted before widespread adoption.

## Electronic supplementary material

Below is the link to the electronic supplementary material.

Supplementary Material 1

## Data Availability

No datasets were generated or analysed during the current study.

## Abbreviations

cHCC-CCA: Combined hepatocellular carcinoma-cholangiocarcinoma
DP: Delayed phase
EASL: European Association for the Study of the Liver
HBA-MRI: Hepatobiliary agent-enhanced magnetic resonance imaging
HBP: Hepatobiliary phase
HCC: Hepatocellular carcinoma
iCCA: Intrahepatic cholangiocarcinoma
KLCA-NCC: Korean Liver Cancer Association-National Cancer Center
LI-RADS: Liver Imaging Reporting and Data System
LT: Liver transplantation, MR, magnetic resonance
PVP: Portal venous phase
TP: Transitional phase
TG: Threshold growth
