# Supplementary material for: Value of threshold growth for the diagnosis of hepatocellular carcinoma using LI-RADS
Source: Cancer Imaging. 2025 Jul 1;25:84. doi: 10.1186/s40644-025-00902-z (PMC12217194; doi:10.1186/s40644-025-00902-z)
Supplement: Supplementary file 1 — Supplementary Material 1 [file 40644_2025_902_MOESM1_ESM.docx]

**Additional file 1**

**Supplementary methods**

*Hepatobiliary agent-enhanced magnetic resonance imaging (MRI) acquisition*

Liver MRI exams were performed on 3.0-T or 1.5-T scanners. The routine liver MRI protocol of our institution includes the following sequences: a respiratory-triggered T2-weighted fast spin-echo sequence, a half-Fourier acquisition single-shot turbo spin-echo sequence, diffusion-weighted imaging, breath-hold T1-weighted gradient-echo in and out-of-phase sequences, and breath-hold T1-weighted fat-suppressed 3D gradient-echo sequences for precontrast and post-contrast imaging including the arterial phase, portal venous phase (PVP), transitional phase, and hepatobiliary phase. MRI scan parameters are described in **Table S1**. For dynamic phase imaging, after obtaining precontrast images, a standard dose (0.025 mmol/kg) of gadoxetic acid (Primovist, Bayer) was injected intravenously at a rate of 1.0 mL/sec using a power injector, followed by a 20-mL saline flush. Using a real-time MRI fluoroscopic monitoring system, arterial phase axial images were acquired 7–8 seconds after contrast material arrival at the distal thoracic aorta. Subsequently, PVP, transitional phase, and hepatobiliary phase axial images were obtained approximately 60 seconds, 3 minutes, and 20 minutes, respectively, after starting the injection of contrast medium.

**Table S1. MRI parameters**

| **Pulse sequence** | **3.0T** |  |  |  |  |  |  |  | **1.5T** |  |  |  |  |  |  |  |
| --- | --- | --- | --- | --- | --- | --- | --- | --- | --- | --- | --- | --- | --- | --- | --- | --- |
|  | TR (msec) | TE (msec) | Flip angle (°) | Slice thickness (mm) | Reconstruction interval (mm) | Image matrix | FOV (mm) | B values (sec/mm^2^) | TR (msec) | TE (msec) | Flip angle (°) | Slice thickness (mm) | Reconstruction interval (mm) | Image matrix | FOV (mm) | B values (sec/mm^2^) |
| T2-weighted FSE | 3000 | 86 | 120 | 6 | 6 | 448×269 | 380×380 | N/A | 2100 | 84 | 90 | 7 | 7 | 256×256 | 380×380 | N/A |
| HASTE | ∞ | 153 | 120 | 6 | 6 | 384×230 | 380×380 | N/A | ∞ | 160 | 90 | 7 | 7 | 320×192 | 380×380 | N/A |
| DWI | 2000 | 62 | 90 | 5 | 5 | 150×120 | 400×320 | 0, 800 | 5000 | 65 | 90 | 5 | 5 | 128×96 | 380×380 | 0, 800 |
| T1-weighted in-/out-of-phase | 4.4 | 2.9/1.5 | 9 | 3 | 3 | 384×307 | 380×380 | N/A | 7.9 | 5.2/2.3 | 12 | 6 | 3 | 320×192 | 380×380 | N/A |
| T1-weighted 3D GRE | 3.6 | 1.4 | 11 | 3 | 3 | 384×384 | 380×380 | N/A | 4.5 | 2.2 | 12 | 6 | 3 | 320×224 | 380×380 | N/A |

Parameters of Skyra 3.0T (Siemens Healthineers) or Signa HDxt 1.5T (GE Healthcare). TR, repetition time; TE, echo time; FOV, field of view; FSE, fast spin-echo; HASTE, half-Fourier acquisition single-shot turbo spin-echo sequence; DWI, diffusion-weighted imaging; GRE, gradient-echo; N/A, not applicable; MRI, magnetic resonance imaging.

**Table S2**. **Results of interval growth rate according to LI-RADS categories**

|  | **LR-1**  **(n = 1)** | **LR-2**  **(n = 2)** | **LR-3**  **(n = 18)** | **LR-4**  **(n = 152)** | **LR-5**  **(n = 88)** | **LR-M**  **(n = 19)** |
| --- | --- | --- | --- | --- | --- | --- |
| No growth | 1 (100.0%) | 1 (50.0%) | 8 (44.4%) | 37 (24.3%) | 9 (10.2%) | 5 (26.3%) |
| Subthreshold growth |  |  |  |  |  |  |
| Growth < 50% |  |  | 6 (33.3%) | 90 (59.2%) | 44 (50.0%) | 8 (42.1%) |
| New lesion |  | 1 (50.0%) | 3 (16.7%) | 20 (13.2%) | 3 (3.4%) |  |
| Threshold growth |  |  | 1 (5.6%) | 5 (3.3%) | 32 (36.4%) | 6 (31.6%) |

Data are reported as numbers (percentages).

LI-RADS, Liver Imaging Reporting and Data System

**Table S3. Diagnostic performance of LI-RADS category 5 (LR-5) for the diagnosis of HCC in subgroups of observations ≤ 2.0 cm and those > 2.0 cm**

|  | **Sensitivity** | **Difference** | **P value** | **Specificity** | **Difference** | **P value** |
| --- | --- | --- | --- | --- | --- | --- |
| Observations ≤ 2.0 cm (n = 214) | | | | | | |
| LI-RADS without TG | 28.3 (21.5, 36.0) | 8.2 (3.9, 12.4) | < 0.001 | 100.0 (93.5, 100.0) | -7.3 (-14.1, -0.4) | 0.125 |
| LI-RADS with TG | 36.5 (29.0, 44.5) |  |  | 92.7 (82.4, 98.0) |  |  |
| Observations > 2.0 cm (n = 66) | | | | | | |
| LI-RADS without TG | 52.1 (37.2, 66.7) | 2.1 (-2.0, 6.1) | > 0.999 | 100.0 (81.5, 100.0) | 0 | Not calculated |
| LI-RADS with TG | 54.2 (39.2, 68.6) |  |  | 100.0 (81.5, 100.0) |  |  |

Data are reported as percentages (95% confidence intervals).

HCC, hepatocellular carcinoma; LI-RADS, Liver Imaging Reporting and Data System; TG, threshold growth
